# Exploring Pyrrolo-Phenanthrolines as Semiconductors for Potential Implementation in Organic Electronics

**DOI:** 10.3390/ma16093366

**Published:** 2023-04-25

**Authors:** Corneliu Doroftei, Liviu Leontie, Ramona Danac, Cristina-Maria Al Matarneh, Aurelian Carlescu

**Affiliations:** 1 Science Research Department, Institute of Interdisciplinary Research, Research Center in Environmental Sciences for the North-Eastern Romanian Region (CERNESIM), Alexandru Ioan Cuza University of Iasi, 11 Carol I, 700506 Iasi, Romania; lleontie@uaic.ro (L.L.); aurelian.carlescu@uaic.ro (A.C.); 2 Faculty of Physics, Alexandru Ioan Cuza University of Iasi, 11 Carol I, 700506 Iasi, Romania; 3 Faculty of Chemistry, Alexandru Ioan Cuza University of Iasi, 11 Carol I, 700506 Iasi, Romania; rdanac@uaic.ro (R.D.); almatarneh.cristina@yahoo.ro (C.-M.A.M.); 4 Institute of Macromolecular Chemistry “Petru Poni”, Aleea Ghica Voda, No. 41A, 700487 Iasi, Romania

**Keywords:** thin organic layers, pyrrolo[1,2-i][1,7]phenanthroline, structural properties, optoelectronic properties, organic thermistor

## Abstract

This paper describes the synthesis and characterization of new organic semiconductors based on pyrrolo[1,2-i][1,7]phenanthrolines in the form of thin layers. The thin layers, produced via the spin coating method (with a thickness of 10–11 μm), were investigated for their electrical and optical properties. After heat treatment at temperatures ranging from 210 to 240 °C, the layers displayed consistent and reproducible properties. The layers exhibited n-type semiconductor behavior, with a thermal activation energy (*E_a_*) in the range of 0.75–0.78 eV. Additionally, the layers showed transmittance values of 84–92% in the visible and near-infrared spectral ranges, with a direct optical band gap (*E_go_^d^*) ranging from 3.13 to 4.11 eV. These thin layers have potential applications in electronic devices such as thermistors, as well as in nanoelectronics and optoelectronics. Overall, these new organic semiconductors show promising properties for practical implementation in various electronic applications.

## 1. Introduction

The use of small molecule organic semiconductors has become increasingly popular in recent years for the development of organic electronics on a larger scale [1]. Organic electronics offers a cost-effective approach for building high-performance devices with large areas, utilizing low-temperature techniques that are compatible with flexible devices, opening up new possibilities for various applications [2,3,4]. Phenanthrolines derivatives are versatile polycyclic organic ligands that have been explored for solid-state device technology, including organic light-emitting diodes (OLEDs), luminescence, and pharmacology [5,6,7,8]. The extended π-conjugated systems of phenanthrolines are particularly interesting due to their large charge transfer through substituent groups on the aromatic rings, making them an ideal nonlinear optical system [5,6,7,8,9]. This class of organic molecules exhibits a significant potential for applications in biochemistry, photochemistry, nonlinear optics, electroluminescence, and other fields [5,10,11].

One subclass of phenanthrolines, pyrrolo-phenanthroline-based organic compounds, proved to be highly processable and offer various coating methods, including printing, evaporation, and centrifugation. However, challenges with uniformity, thickness control, and transformation prevention during coating may hinder printing and evaporation methods. Fortunately, centrifugation coating surpasses these limitations and boasts superior advantages. Remarkably, the spin coating technique, which was originally developed for coating silicon layers with photoresist in the microelectronics industry, has demonstrated remarkable efficacy for coating organic compounds.

In the above context and as a continuation of our work in the field of phenanthroline compounds [7,8,10,11], in this study we introduce a series of organic compounds based on thepyrrolo[1,2-i][1,7]phenanthroline skeleton, which we synthesized and characterized for their structural, electrical, and optical properties as thin layers, using the spin coating technique. Notably, we also explore the potential of these organic thin layers for use as negative temperature coefficient (NTC) thermistors.

## 2. Materials and Methods

### 2.1. Synthesis of Organic Compounds Based on Pyrrolo[1,2-i][1,7] Phenanthrolines

New indolizine derivatives with the phenanthroline skeleton PP/CHN-1, PP/CHN-2, and PP/CHN-3 were obtained using Huisgen [3+2] dipolar cycloaddition reactions, where 1,7-phenanthrolin-7-ium N-ylides 2(a-c) were generated in situ from the corresponding mono-salts 1(a–c) in basic medium (Et_3_N).

**Figure 1:**
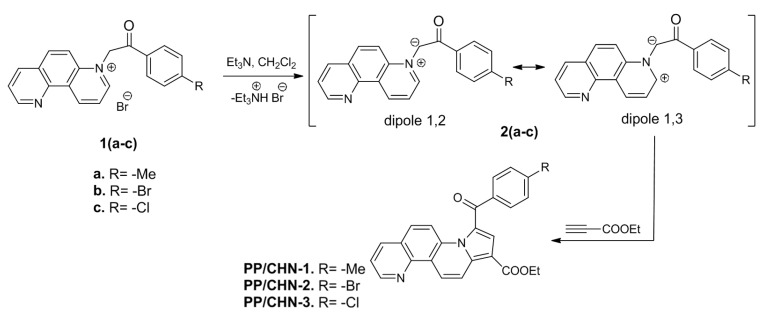
Synthesis pathway of the studied organic compounds, type PP/CHN.

The generated N-ylides, acting as 1,3-dipoles, were reacted with ethyl propiolate (as dipolarophile) to produce the desired PP/CHN compounds (Figure 1) [6,12,13].

The synthesized compounds were obtained as polycrystalline powders and exhibited good stability of the chemical structure under a normal ambient atmosphere.

The structure of the compounds (including the nature of substituents) is given, together with the corresponding melting points, in Table 1. These products were characterized by using spectral (IR, ^1^H-, and ^13^C-Nuclear Magnetic Resonance and mass spectrometry (MS)) and analytical methods [12,13,14,15,16,17,18,19].

In the IR spectra of the compounds PP/CHN (1–3), the absorption band related to the ester group C=O appears in the range of 1714–1692 cm^−1^, while the aromatic ketone groups absorb in the range of 1626–1631 cm^−1^. The vibrations of C-O ester bonds produce intense absorption bands in the specific area of 1236–1077 cm^−1^.

The characteristic NMR signals of the studied compounds confirm the structure of the synthesized compounds and are presented in Table 2.

In addition, the structures of the compounds PP/CHN (1–3) were proven by using MALDI-TOF MS, which confirmed their exact masses as indicated in Table 3.

### 2.2. Obtaining and Techniques Used for the Characterization of Compounds and Organic Thin Layers

Proton and carbon nuclear magnetic resonance spectra were recorded on a Bruker Avance 400 DRX (400 MHz) or a Bruker ARX (300 MHz).

IR spectra were recorded on a FTIR Shimadzu spectrophotometer, using KBr pelletes of the investigated compounds.

Mass spectra were acquired on a Bruker RapifleX MALDI-TOF/TOF (Bruker Daltonics, Bremen-Germany) equipped with a Smartbeam 3D laser. The FlexControl Version 4.0 and FlexAnalysis Version 4.0 software (Bruker, Bremen, Germany) were used to control the instrument and process the MS spectra using the following parameters: positive ion polarity in reflector mode, mass scan range *m*/*z* 100–1600 Da, digitizer 1.25 GHz, detector voltage 2117 V, 1000 shots per pixel, and 5 kHz laser frequency. The laser power was set at 60% to 80% of the maximum and 1000 laser shots were accumulated for each spectrum.

Synthesized organic pyrrolo[1,2-*i*][1,7]phenanthroline compounds, in the form of powders, were dissolved in chloroform in order to obtain the corresponding solutions with a concentration in the range of 2–10 mg/mL.These solutions were then used to form thin layers using the spin coating deposition technique [20,21,22,23,24], which offers several benefits such as compatibility with different substrates and coating solutions, precise control over film thickness, and good reproducibility [21,25,26,27]. The layers were deposited on transparent amorphous glass substrates with dimensions of 10 × 10 × 1 mm^3^ at a rotation of the substrate of 1500 rpm (static variant), with 5–8 cycles of coating (20 °C/40% RH) and drying (50–60 °C). After deposition, the layers were subjected to a heat treatment at a temperature 10 °C lower than the melting point of the corresponding compound (Table 1) for a duration of 30 min.

Determinations regarding the thickness of the layers were made by using interferometric microscopy. The microstructural and morphological properties were determined by means of X-ray diffractometry (XRD) and atomic force microscopy (AFM) measurements, respectively.

The study of the optical properties of the organic layers deposited on glass substrates was carried out in the spectral range of 230–1700 nm using an optical spectrometer operating in the ultraviolet–visible–near-infrared (UV–VIS–NIR) range.

The layers deposited on glass substrates with previously deposited silver electrodes were subjected to a study of their d.c. electrical properties. The semiconductor behavior of the layers was determined through Hall measurements (four-probe method), while their temperature dependence of electrical conductivity was determined via electrical measurements (two-probe method). These measurements were carried out using an RLC measuring bridge and an electric heating device equipped with electrical contacts for electrodes.

## 3. Results and Discussion

**Figure 2:**
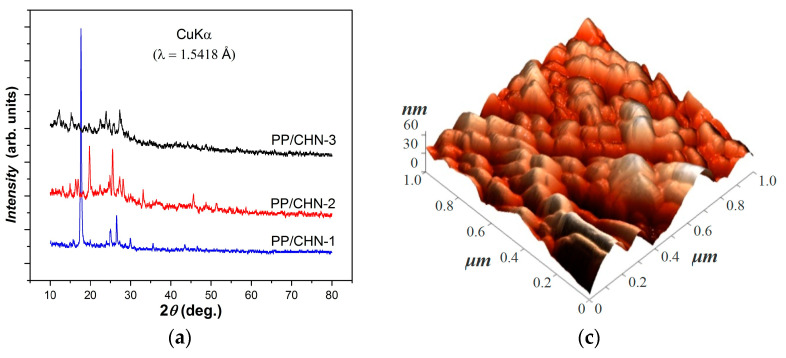

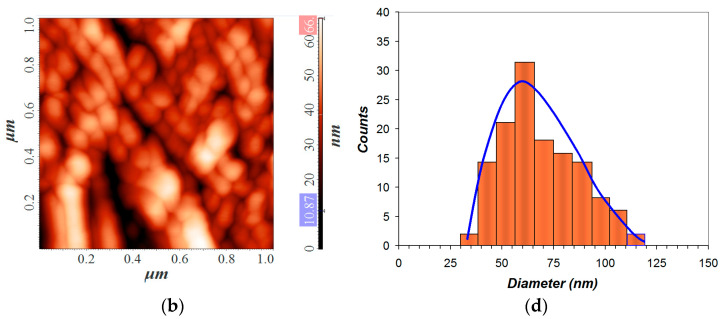
XRD patterns for all three organic layers studied: (**a**) typical 2D and 3D AFM image (**b**,**c**) and the histogram showing the height profiles of the AFM images (**d**) for the PP/CHN-1 layer made at a scale of 1.0 × 1.0 µm^2^.

Our XRD investigations revealed differences in the polycrystalline structure of the organic layers, depending on the substituent R of the organic molecules. Specifically, we found that derivatives PP/CHN-1, PP/CHN-2, and PP/CHN-3 with R = −Me, R = −Br, and R = −Cl, respectively, exhibited distinct XRD patterns (Figure 2a) at room temperature. The relevant structural parameters resulting from our analysis are summarized in Table 4.

The polycrystalline layers we obtained exhibited distinct structures depending on the substituent R. However, the interplanar distances (*d*) (Table 4) shared certain values due to the cyclic skeleton of the constituent molecules. These layers consisted of nanometric crystals, with average crystallite sizes that increased slightly from 28.48 nm (in the case of PP/CHN-1) to 30.11 nm (in the case of PP/CHN-2), and to 39.92 nm (for PP/CHN-3). The thicknesses of the layers were similar, ranging from 11 to 12 µm (Table 4), and their morphology did not vary significantly across different structures. The layers exhibited a clustered structure with uniform particle agglomerations of 30–120 nm (Figure 2b–d) and an average roughness (*Ra*) of approximately 55 nm.

The electrical properties of materials in the form of thin layers can differ greatly from those of bulk materials [33]. In the case of polycrystalline thin layers, the electrical conductivity is typically lower, but the mobility of the charge carriers is higher. This is due to scattering mechanisms that occur on the sample’s surface and at the boundaries of its crystallites [34,35].

**Figure 3:**
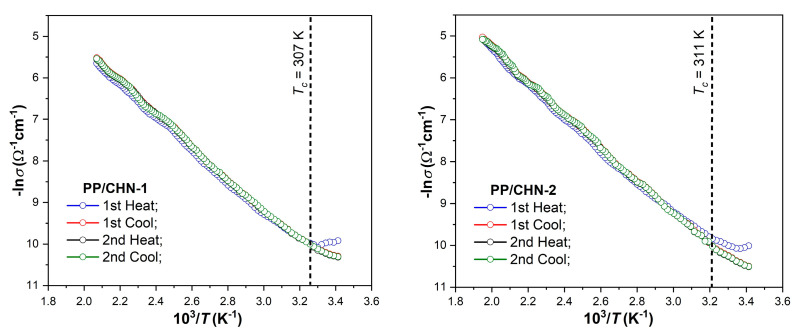

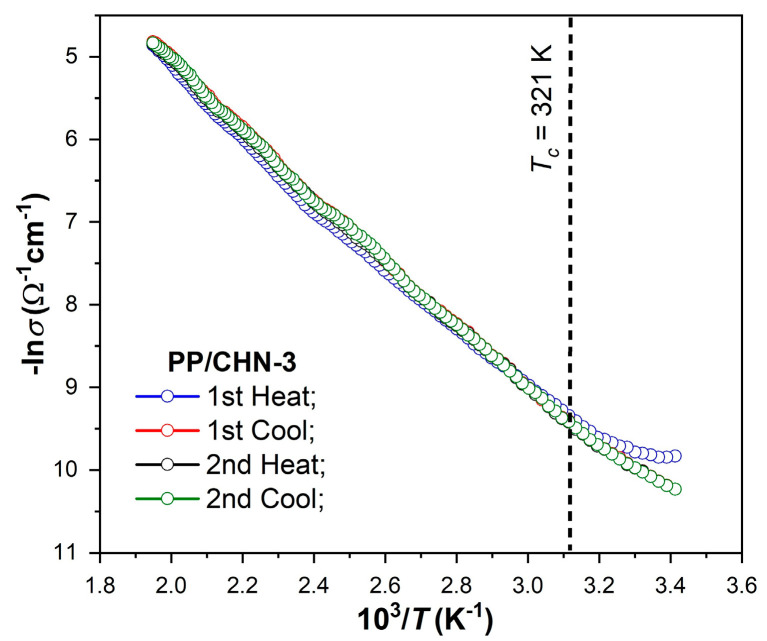
The dependence ln*σ* vs. 1000/*T* for two heating–cooling cycles of the studied organic layers.

The electrical conductivity of the layers (*σ*) as a function of temperature was determined for several heating–cooling cycles, confirming the reproducibility of the characteristics and therefore the good stability of the layers in the temperature range Δ*T*, at which the measurements were made (Table 5). The temperature-dependent conductivities of the organic layers [ln*σ* = f(1000/*T*)] for two heating–cooling cycles are presented in Figure 3. According to the band model representation, the conductivity–temperature dependences show two conduction regions (with different slopes) delimited by a certain value of the temperature (*T_c_*), specific to the respective organic layer. Therefore, for the lower temperature range (*T < Tc*), extrinsic conduction takes place, whereas for the higher temperature range (*T > Tc*), intrinsic conduction occurs.

Using the slope of the ln*σ* = f(1000/*T*) curve (its linear portion), the value of the thermal activation energy of electrical conduction (*Ea*) can be determined. Displaying two conduction regions, the layers exhibit two thermal activation energies involving different electrical conduction mechanisms, depending on the respective temperature range. These characteristics are typical of wide-band semiconductors.

The activation energy, *Ea*, was determined for the intrinsic conduction region and according to the band model, its value represents half of the band gap value (*E_g_*) of the respective compound (Table 5) [36].

Hall measurements were conducted to determine the semiconductor behavior of the layers, which revealed that these organic semiconductors exhibit n-type behavior, where electrons are the dominant charge carriers.

The organic semiconductors being studied herein contain small molecules with aromatic groups, which results in the presence of highly delocalized π electrons along the molecular backbone. The increase in the number of *π* electrons leads to the decrease in the thermal activation energy and to the increase in the electrical conductivity [37,38]. In addition, the value of the thermal activation energy can vary depending on the position and nature of the substituents R in the molecules; they influence the transfer of electrons inside the molecules due to the presence of the conjugation systems in the studied organic layers (Figure 1). The compound PP/CHN-1, which contains a methyl R-substituent, had the lowest thermal activation energy due to the electron-donating effect of the methyl group. On the other hand, the other two compounds, which have bromine or chlorine substituents in the *para* position of the phenyl ring, exhibited similar values of thermal activation energy. This similarity is likely due to the similar electronic behavior of bromine and chlorine.

**Figure 4:**
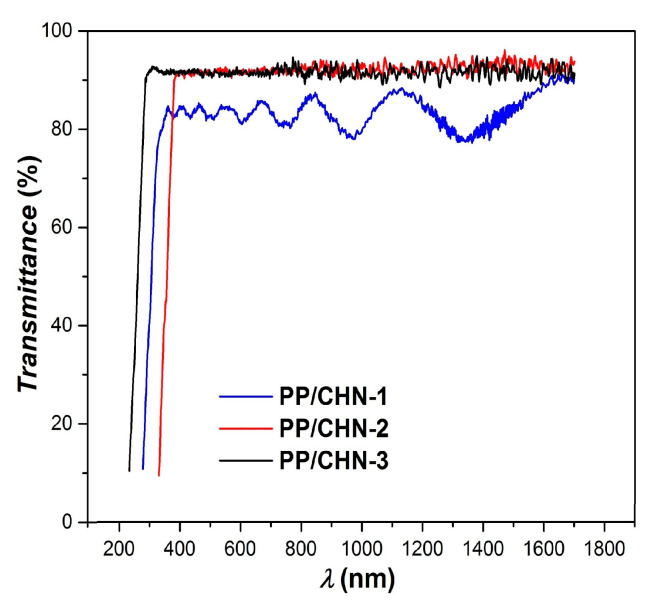
The transmittance spectra of the organic layers under study.

The investigations on the optical properties of the obtained organic layers were carried out in the spectral range between 230 and 1700 nm (UV–VIS–NIR). Figure 4 shows the spectral (wavelength, λ) dependence of transmittance (*T*) for the studied layers. At lower layer thicknesses, as shown by the PP-CHN-2 and PP-CHN-3 layers, an attenuation of the interference maxima and minima is observed, as well as a higher transmission, in accordance with Beer–Lambert law [39,40]:

(1) $$T=e^{–\alpha \delta }$$

where *α* is the absorption coefficient of the layer and *δ* denotes its thickness.

In the visible and near-infrared spectral ranges, the PP-CHN-1 layer exhibits a transmittance of 84%, while that of the PP-CHN-2 and PP-CHN-3 layers is of 92%. In addition, the reflectance (*R*) values are below 16% for the PP-CHN-1 layer and below 8% for the PP-CHN-2 and PP-CHN-3 layers.

By investigating the absorption of electromagnetic radiation in semiconductors, information can be obtained regarding the structure of energy bands, the type of optical transitions, the width of the forbidden band, etc.

The absorption coefficient (α) was determined from the following relation [41]:

(2) $$\alpha =\frac{1}{\delta }\ln(1–R^{2})/T$$

where *δ* represents the thickness, *T* denotes the transmittance, and *R* is the reflectance of the layer.

**Figure 5:**
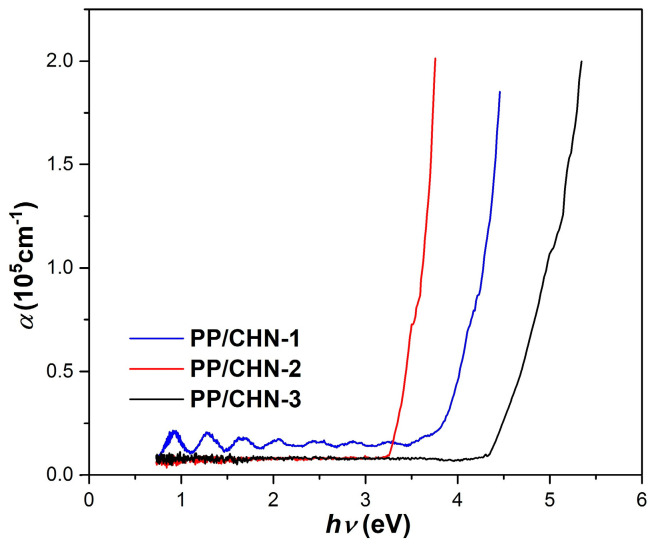
The optical absorption spectra of the examined organic layers.

The absorption spectra, which show the absorption coefficient (α) as a function of the incident photon energy (*hν*), of the organic layers are displayed in Figure 5. It can be observed that the absorbance for all the layers studied increases with an increase in photon energy. The PP-CHN-1 layer shows the greatest increase in absorbance in the UV–VIS region (3.79–4.45 eV), while the PP-CHN-2 layer, in the VIS region (3.23–3.75 eV) and the PP-CHN-3 layer, in the UV region (4.33–5.34 eV).

The relation in [42,43] is as follows:

(3) $$\alpha h\nu =A{\left(h\nu –E_{g0}\right)}^{n}$$

This expresses the energy (*hν*) dependence of the absorption coefficient (*α*) at the limit of the absorption edge, which can be expressed as follows [41,44]:

(4) $$\frac{d[\ln(\alpha h\nu )]}{d[h\nu ]}=\frac{n}{h\nu –E_{g0}}$$

where *A* is a factor that does not depend on *hν*, *n* is a constant that has the value of one half for direct optical transitions and two for allowed indirect optical transitions, and *E_go_* is the optical band gap (*E_go_^d^*—director *E_go_^i^*—indirect).

**Figure 6:**
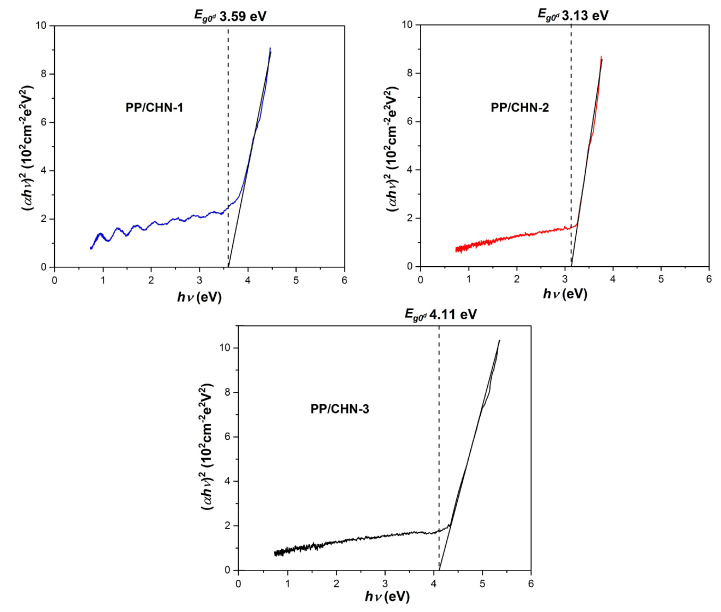
Optical absorption spectra of organic compounds under study, illustrating the direct band gaps.

The optical band gap (*E_go_*) was determined from the graphic representation (*αhν*)^2^ as a function of *hν* (Figure 6) by extrapolating the linear region of the graph at zero absorption. The values obtained for the direct band gaps (*E_go_^d^*) were 3.59 eV for the PP-CHN-1 layer, 3.13 eV for the PP-CHN-2 layer, and 4.11 eV for the PP-CHN-3 layer.

The thermal activation energy (*E_a_*) values obtained through electrical measurements differ from the optical band gap energy (*E_go_*) values obtained through optical measurements due to the distinct nature of the carrier excitation during the processes of electrical conduction and optical absorption, respectively.

Semiconducting organic layers have also been investigated from the point of view of potential applications in organic thermistors with a negative temperature coefficient, in which the electrical resistance decreases with increasing temperature. In general, NTC thermistors are used as current limiters and temperature sensors.

The electrical resistance of a semiconductor as a function of its absolute temperature in the intrinsic conduction regime can be expressed by the following relation [45]:

(5) $$R(T)=R_{0}e^{\frac{B}{T}}$$

where *R*(*T*) represents the electrical resistance of the semiconductor at temperature *T*, *R*_0_ is the resistance at infinite temperature, and *B* is a temperature sensitivity parameter of the semiconductor, given by the following relation [45,46]:

(6) $$B=\Delta E/2k_{B}$$

where Δ*E* is the energy band gap of the semiconductor and *k_B_* is the Boltzmann constant.

The temperature coefficient (*α_T_*)of the semiconductor resistance is defined by the following relation:

(7) $$\alpha_{T}=\frac{1}{R}\frac{dR}{dT}$$

Using relations (5) and (6), the temperature coefficient of the thermistor resistance can be expressed as follows:

(8) $$\alpha_{T}=–\frac{B}{T^{2}}$$

Table 6 shows the values obtained for these two parameters, *B* and *α_T_* (at the reference temperature of 385 K), that characterize the organic semiconductor layers in terms of thermistor properties.

The values obtained in this study are similar to those reported by other researchers who have investigated various semiconductor materials for potential thermistor applications. For instance, Nagai et al. [47] conducted a study on SiC thin layers for NTC thermistor applications and reported a temperature sensitivity parameter (*B*) value of 3080 K at 250 °C. In addition, Jartap et al. [45] conducted a study on a range of thick inorganic layers, consisting of the spinel compound Mn_1.85_Co_0.8_Ni_0.35_O_4_, for NTC thermistor applications. They reported temperature sensitivity parameter (*B*) values ranging from 4014 K to 4223 K at 300 °C.

In addition, it can be emphasized that the values of the temperature sensitivity parameter of the organic semiconductors studied by us are comparable to the catalog values of some commercial NTC thermistors based on oxide semiconductors [NTC Thermistors—produced by Littelfuse, Inc. (USA), symbol: 103JG1F (*B* = 3892 K), symbol: GT103J1K (*B* = 3977 K) and symbol: GP104LBF (*B* = 4040 K)] [48].

Comparing these results, it can be concluded that the investigated organic semiconductors, in the form of thin layers, have the potential for use in NTC thermistors.

## 4. Conclusions

This work presents the synthesis of new organic semiconductors based on pyrrolo[1,2-i][1,7]phenanthrolines in the form of thin layers, with the substituents R of the organic molecules R= −Me, R= −Br and R= −Cl, as well as their investigation from the point of view of electrical and optical properties. The layers were deposited on transparent glass by the spin coating method, with thicknesses between 10 and 11 μm. These display a polycrystalline structure and an n-type semiconductor behavior in the studied temperature range between 20 and 210 °C for the organic layer PP/CHN-1 (R= −Me) and between 20 and 240 °C for the organic layers PP/CHN-2 (R= −Br) and PP/CHN-3 (R= −Cl). The examined organic layers exhibit thermal activation energy (*E_a_*) values between 0.75 and 0.78 eV, typical for semiconductor materials. In the visible and near-infrared spectral ranges, the layers show transmittance values of 84–92% and a direct optical band gap (*E_go_^d^*) in the range of 3.13–4.11 eV. We found that these organic layers have potential applications in NTC thermistors, with temperature sensitivity parameter (*B*) values in the range of 3868–3928 K at a temperature of 385 K. Furthermore, our results suggest that these compounds in the form of thin layers can be used in various organic nanoelectronics and optoelectronics applications.

## Figures and Tables

**Table 1:** Physico-chemical properties of the studied organic compounds.

Compound	PP/CHN-1	PP/CHN-2	PP/CHN-3
Chemical formula	C_26_H_20_N_2_O_3_	C_25_H_17_BrN_2_O_3_	C_25_H_17_ClN_2_O_3_
Molecular weight (M(g/mol))	408.45	473.72	428.87
Color	orange	yellow	yellow
Melting point (°C)	219–221	250–251	253–255

**Table 2:** NMR chemical shifts of hydrogen and carbon atoms of the compounds PP/CHN (1–3).

Compound	Aliphatic H	Aromatic H	Aliphatic C	Aromatic C	Ester C	Ketone C
	Chemical shift (CDCl_3_, δ (ppm))					
PP/CHN-1	1.31 (CH_3_) 2.43 (CH_3_) 4.33 (CH_2_)	7.31, 7.48, 7.68, 7.81, 7.97, 8.04, 8,18, 8.50, 8.99, 9.16	14.5 (CH_3_) 21.7 (CH_3_) 60.3 (CH_2_)	107.6, 118.6, 121.0, 121.8, 124.4, 125.4, 127.6, 127.9, 129.3, 130.3, 133.9, 135.5, 137.7, 140.7, 143.9, 149.4	164.0	184.4
PP/CHN-2	1.43 (CH_3_) 4.41 (CH_2_)	7.58, 7.73, 7.76, 7.94, 8,00, 8.14, 8.27, 8.60, 9.09, 9.30	14.6 (CH_3_) 60.3 (CH_2_)	107.6, 118.1, 120.3, 122.0, 122.8, 125.4, 125.3, 127.2, 127.9, 128.0, 131.5, 131.9, 130.7, 133.6, 136.1, 137.2, 141.4, 145.2, 150.5	163.9	83.1
PP/CHN-3	1.42 (CH_3_) 4.42 (CH_2_)	7.58, 7.78, 7.94, 8.10, 8.14, 8.33, 8.62, 9.12, 9.33	14.5 (CH_3_) 60.4 (CH_2_)	107.8, 118.3, 122.1, 122.0, 125.1, 125.4, 127.2, 127.8, 128.9, 131.4, 130.7, 133.8, 136.7, 139.4, 141.2, 144.2, 149.8	163.8	183.0

**Table 3:** Calculated and experimental MS data of the compounds PP/CHN (1–3).

Compound	Calculated Exact Mass (*m*/*z*)	Experimental Exact Mass (*m*/*z*)
PP/CHN-1 (C_26_H_20_N_2_O_3_ + H)^+^	409.16	409.14
PP/CHN-2 (C_25_H_17_BrN_2_O_3_ + H)^+^	473.05	473.01
PP/CHN-3 (C_25_H_17_ClN_2_O_3_ + H)^+^	429.10	429.09

**Table 4:** Structural parameters determined from the analysis of XRD patterns.

PP/CHN-1					PP/CHN-2					PP/CHN-3				
*δ* (μm)	*I/I*_0_ (%)	2*θ* (deg.)	*d* (nm)	*D* (nm)	*δ* (μm)	*I/I*_0_ (%)	2*θ* (deg.)	*d* (nm)	*D* (nm)	*δ* (μm)	*I/I*_0_ (%)	2*θ* (deg.)	*d* (nm)	*D* (nm)
	51.19	14.93	0.593	29.91		86.80	10.56	0.837	21.94		96.96	11.05	0.800	30.89
	51.81	15.74	0.563	26.19		86.37	10.76	0.822	55.60		100.00	12.32	0.718	14.91
	100.00	17.66	0.502	30.01		86.80	11.43	0.774	37.93		96.96	13.28	0.666	96.96
	51.81	19.94	0.445	21.61		87.53	13.15	0.673	26.96		96.20	13.87	0.638	96.20
	51.50	23.97	0.371	31.44		85.53	14.12	0.627	30.98		99.24	15.24	0.581	15.23
	54.20	25.01	0.356	25.01		88.38	14.92	0.593	27.91		96.20	17.17	0.516	15.26
12	57.29	26.52	0.336	31.59		89.65	16.36	0.541	29.96		95.54	18.57	0.477	15.29
	51.50	27.27	0.327	31.64		89.65	16.97	0.522	23.32	11	96.20	19.60	0.452	31.21
	52.12	29.91	0.298	28.64		85.53	17.64	0.502	32.31		95.16	20.99	0.423	95.16
	50.88	35.55	0.252	31.14	11	85.95	18.21	0.487	15.29		97.34	22.43	0.396	30.23
	49.96	39.19	0.229	31.47		100.00	19.80	0.448	30.10		99.62	23.87	0.372	27.37
	50.57	43.45	0.208	18.24		87.96	22.43	0.396	23.51		97.34	24.90	0.357	31.48
	50.27	45.52	0.195	33.47		88.80	24.51	0.363	44.72		96.20	25.90	0.344	15.49
						90.91	24.95	0.356	31.49		100.00	27.29	0.326	15.53
						99.15	25.54	0.348	31.53		91.09	44.21	0.204	91.09
						90.49	27.29	0.326	17.80		90.33	48.72	0.186	16.57
						89.65	28.12	0.317	19.02					
						86.37	33.07	0.270	26.24					
						85.11	45.62	0.198	33.36					
						82.57	48.70	0.186	39.62					
						82.15	51.31	0.178	32.89					

*δ*—layer thicknesses; *I*/*I*_0—_relative intensity (*I*_0—_maximum peak intensity); *θ*—Bragg diffraction angle; *d*—interplanar distance determined from the Bragg equation, 2*d*sin*θ* = *nλ* [28,29], for the order of reflection *n* = 1 and the X-ray wavelength *λ* = 1.5418 Ǻ; *D*—average crystallite size determined from the Scherrer equation, *D* = 0.9*λ*/*β*cos*θ* [30,31,32], where β is the full-width at half-maximum of the diffraction peak.

**Table 5:** The values of the electrical transport parameters for the studied organic layers.

Sample	*σ_c_* (Ω^−1^·cm^−1^)	Δ*T* (K)	*σ_T_* (Ω^−1^·cm^−1^)	*T_c_* (K)	*E_a_* (eV)	*E_g_* (eV)
PP/CHN-1	1.99 × 10^−4^	293–483	3.93 × 10^−3^	307	0.758	1.516
PP/CHN-2	1.27 × 10^−4^	293–513	6.20 × 10^−3^	311	0.785	1.570
PP/CHN-3	1.45 × 10^−4^	293–513	7.93 × 10^−3^	321	0.782	1.564

*σ_c_*—electrical conductivity before the heat treatment; *σ_T_*—electrical conductivity at the upper limit of temperature range.

**Table 6:** The values of the *B* and *α_T_* parameters for the studied organic semiconductor layers.

Sample	*B* (K)	*−α_T_* (K^−1^)
PP/CHN-1	3928	0.0265
PP/CHN-2	3879	0.0262
PP/CHN-3	3867	0.0261

## Data Availability

The data presented in this study are available on request from the corresponding author.
